# Multimodal classification of mood disorders using structural MRI and polygenic risk scores: a machine learning ablation study in UK Biobank

**DOI:** 10.3389/fpsyt.2026.1883424

**Published:** 2026-07-27

**Authors:** Umi Kalsom Mohamad Yusof, Joey Ward, Laura M. Lyall, Rona J. Strawbridge

**Affiliations:** 1 School of Health and Wellbeing, University of Glasgow, Glasgow, United Kingdom; 2 Centre for Clinical Brain Sciences, University of Edinburgh, Edinburgh, United Kingdom

**Keywords:** data fusion, major depressive disorder, mania/bipolar disorder, multimodal machine learning, neuroimaging, polygenic risk scores, UK Biobank

## Abstract

**Objective:**

Mood disorder classification from neuroimaging alone remains limited by single-modality constraints and population-level heterogeneity. This study proposes a fusion-based machine learning framework combining structural brain MRI and polygenic risk scores (PRS) derived from genome-wide association studies, alongside demographic and imaging confounder data, to classify individuals into three categories: healthy controls (HC), mania/bipolar disorder (MBD), and major depressive disorder (MDD).

**Methods:**

Data were drawn from the UK Biobank under a retrospective, cross-sectional design. Three dataset configurations were structured as an ablation study: neuroimaging-only (D1; n=426), genetics-only (D2; n=264), and full multimodal fusion (D3; n=264). T1-weighted 3D MRI volumes were converted to grayscale, and the eight axial slices with the highest Shannon entropy per participant were selected, resized to 64×64 pixels, and reduced from 32,768 to 150 features via ANOVA F-test univariate selection. PRS for bipolar disorder and MDD were derived from Psychiatric Genetics Consortium GWAS summary statistics using LDpred, restricted to European ancestry participants. Six machine learning classifiers were trained under each configuration using an 80:20 stratified split with 10-fold cross-validation, with macro-averaged AUC as the primary metric.

**Results:**

XGBoost achieved the strongest discriminatory performance, attaining a macro-average AUC of 0.7647 and an MDD-specific AUC of 0.8175 on the neuroimaging-only configuration, representing peak performance across all conditions. The genetics-only configuration produced substantially lower macro-average AUC values across all classifiers (XGBoost: 0.6600), consistent with the modest individual-level effect sizes of common psychiatric genetic variants. The controlled same-sample comparison between D2 and D3 revealed that the addition of MRI features improved macro-average AUC by 0.052 (XGBoost: 0.6600 to 0.7121), with the most pronounced gain observed for MBD classification (AUC 0.7160 to 0.7840). Despite its smaller sample size, D3 achieved competitive performance, suggesting that PRS may provide complementary signal, although direct comparison with D1 is limited by differences in sample composition.

**Conclusion:**

These findings support the potential of interpretable, fusion-based machine learning for advancing diagnostic precision in mood disorder research, with multimodal integration of neuroimaging and genetic risk offering complementary signal beyond what either modality provides independently.

## 1 Introduction

Mood disorders, including major depressive disorder (MDD) and bipolar disorder (BD), represent a significant and growing global health burden. MDD carries a lifetime prevalence of 5 to 17 percent, with an average of around 12 percent (1), while the aggregate lifetime prevalence of BD is estimated at 2.4 percent (2). Despite their prevalence, objective and reliable tools for distinguishing between these conditions and from healthy controls remain limited in clinical practice. Diagnosis continues to rely predominantly on clinical interview and symptom-based criteria, which are susceptible to subjectivity and overlap between conditions. This diagnostic ambiguity contributes to delays in treatment, misclassification, and suboptimal patient outcomes. Accurate differentiation between MDD and BD is of substantial clinical importance, as the two conditions require markedly different pharmacological management; misclassification, particularly the under-recognition of bipolar depression as unipolar MDD, can lead to inappropriate antidepressant monotherapy and is associated with poorer long-term outcomes, including increased risk of mood destabilisation (1, 2).

The integration of diverse medical data modalities into machine learning (ML) predictive models has emerged as a promising avenue for improving diagnostic precision in psychiatry, as well as understanding wider differences between MDD, BD, and healthy individuals (3–5). In the past decade, digitisation of health data has grown substantially across healthcare sectors, encompassing electronic health records (EHR), medical imaging, multi-omics data, and environmental variables (6, 7). Integrating these data sources has become increasingly important for enhancing prediction, diagnosis, and treatment planning, as each modality captures aspects of a patient’s health not fully represented by any single source. In Alzheimer’s disease diagnosis, combining imaging data with cognitive assessments and demographic variables has consistently outperformed single-source models (8). Similar advantages have been reported in diabetic retinopathy prediction (5) and acute respiratory failure detection from chest X-rays and EHR data (4), indicating the broader applicability of multimodal fusion across medical imaging tasks.

Structural brain MRI provides objective measures of brain morphology that differ across psychiatric conditions, while polygenic risk scores (PRS) derived from genome-wide association studies (GWAS) capture inherited genetic liability for specific disorders (6). These two modalities offer complementary information: neuroimaging reflects structural differences between MDD, BD, and healthy control groups, including some which may reflect consequences of illness, while genetic risk scores reflect predisposition independent of disease stage or treatment history. The combination of these modalities holds particular promise for mood disorder classification, yet their systematic integration remains underexplored in the psychiatric ML literature.

The classification of neuropsychiatric disorders using multimodal ML has attracted growing attention. Liu et al. (9) developed an enhanced multi-modal graph convolutional network to classify patients with neuropsychiatric disorders and healthy controls by fusing structural and functional brain graphs, achieving 93.71% accuracy on an open benchmark dataset (9). Rahaman et al. (10) proposed a multimodal classification framework combining structural MRI, functional MRI, and genomic data for schizophrenia prediction, with intermediate fusion incorporating attention mechanisms achieving 92% accuracy (10). In the context of mood disorders, Li et al. (11) combined structural and functional MRI to train a support vector machine classifier for bipolar disorder, using features from voxel-based morphometry and regional homogeneity analyses to achieve 87.5% accuracy (11). Sun et al. (12) proposed an encoder-decoder framework integrating MRI and EHR data for three-class prediction of Alzheimer’s disease and diabetes mellitus, reporting accuracies of 85.9% and 89.9% respectively (12). More recent work has extended multimodal classification further, with He et al. (13) achieving 91.2% accuracy in distinguishing BD from MDD using structural MRI morphometric features combined with a two-stage deep neural network and SVM framework (13), and Cao et al. (14) extending classification to a four-class task incorporating schizophrenia alongside BD, MDD, and HC using 272 cortical features from 68 brain regions (14). Rokham et al. (15) proposed an ensemble deep multimodal framework combining structural and functional MRI for simultaneous mood and psychosis classification (15). In a study closely related to the present work, Lee et al. (16) integrated structural MRI with PRS and whole-exome sequencing for three-class mood disorder classification, reporting that MRI-genetic fusion substantially outperformed genetics-only models with an AUC improvement exceeding 0.21 (16). A related study by Aziz et al. (17) similarly combined structural MRI features with genetic data, using individual single-nucleotide polymorphisms rather than a derived polygenic risk score, for three-class classification of MDD, BD, and healthy controls in a clinical cohort, achieving a peak accuracy of 74.2% using a linear SVM classifier on fused deep MRI features and SNP data (17). Poirot et al. (18) trained machine learning and deep learning classifiers for MDD prediction from T1-weighted structural MRI in nearly 5,000 UK Biobank participants, achieving an AUC of 0.57 from grey matter alone (18), underscoring the limitations of single-modality neuroimaging for population-level mood disorder classification and providing direct motivation for multimodal fusion approaches.

Data fusion strategies in ML are broadly categorised as early fusion, intermediate fusion, and late fusion (6). Early fusion integrates raw features from multiple modalities prior to model training, creating a unified input representation. Intermediate fusion combines modality-specific learned representations at intermediate model layers, while late fusion aggregates predictions from separate modality-specific models at the output level (10). For tabular genetic data combined with image-derived features, early fusion offers a practical and interpretable approach that avoids the added complexity of deep multimodal architectures. Selecting informative slices from 3D MRI volumes is a further practical consideration when training 2D classification models. Hon and Khan (19) proposed selecting slices based on Shannon entropy as a principled alternative to random extraction, arguing that entropy captures the informational richness of each slice and identifies the most structurally complex brain regions (19). Yagis et al. (20) extended this approach by retaining the eight axial slices with the highest entropy per volume for brain MRI classification (20), and Carcagni et al. (21) applied the same strategy in Alzheimer’s disease classification across ordered groups of 4, 8, and 16 slices while ensuring subject-specific slices appeared exclusively in training or validation sets to prevent data leakage (21). Velazquez and Lee further demonstrated the utility of combining diffusion tensor imaging with EHR data for Alzheimer’s disease classification, achieving 98.81% accuracy under a late fusion strategy (22).

Most existing studies frame classification as a binary task, typically distinguishing a single disorder from HC, and studies reporting the highest accuracies operate under binary settings using curated open datasets rather than population cohorts. Three-class simultaneous classification of HC, BD, and MDD from a general population cohort is a substantially more difficult problem, and directly comparable studies remain scarce. While neuroimaging modalities have been widely combined, the integration of PRS with structural MRI for mood disorder classification specifically remains underexplored, with systematic evaluation receiving limited attention outside of Lee et al. (2024) (16). Population-based cohorts such as UK Biobank introduce realistic challenges absent from clinical convenience samples, including greater demographic heterogeneity, smaller diagnostic effect sizes, and constrained case numbers relative to controls.

This study proposes a fusion-based machine learning framework that integrates structural MRI features, polygenic risk scores, and demographic variables to classify individuals from the UK Biobank into HC, mania/bipolar disorder (MBD), and MDD categories. Three dataset configurations are evaluated as an ablation study to isolate the independent and combined contributions of neuroimaging and genetic data to classification performance. To the best of our knowledge, this is among the first studies to systematically evaluate the combined contribution of raw structural MRI features and PRS for simultaneous three-class mood disorder classification in a large population cohort.

## 2 Materials and methods

**Figure 1:**
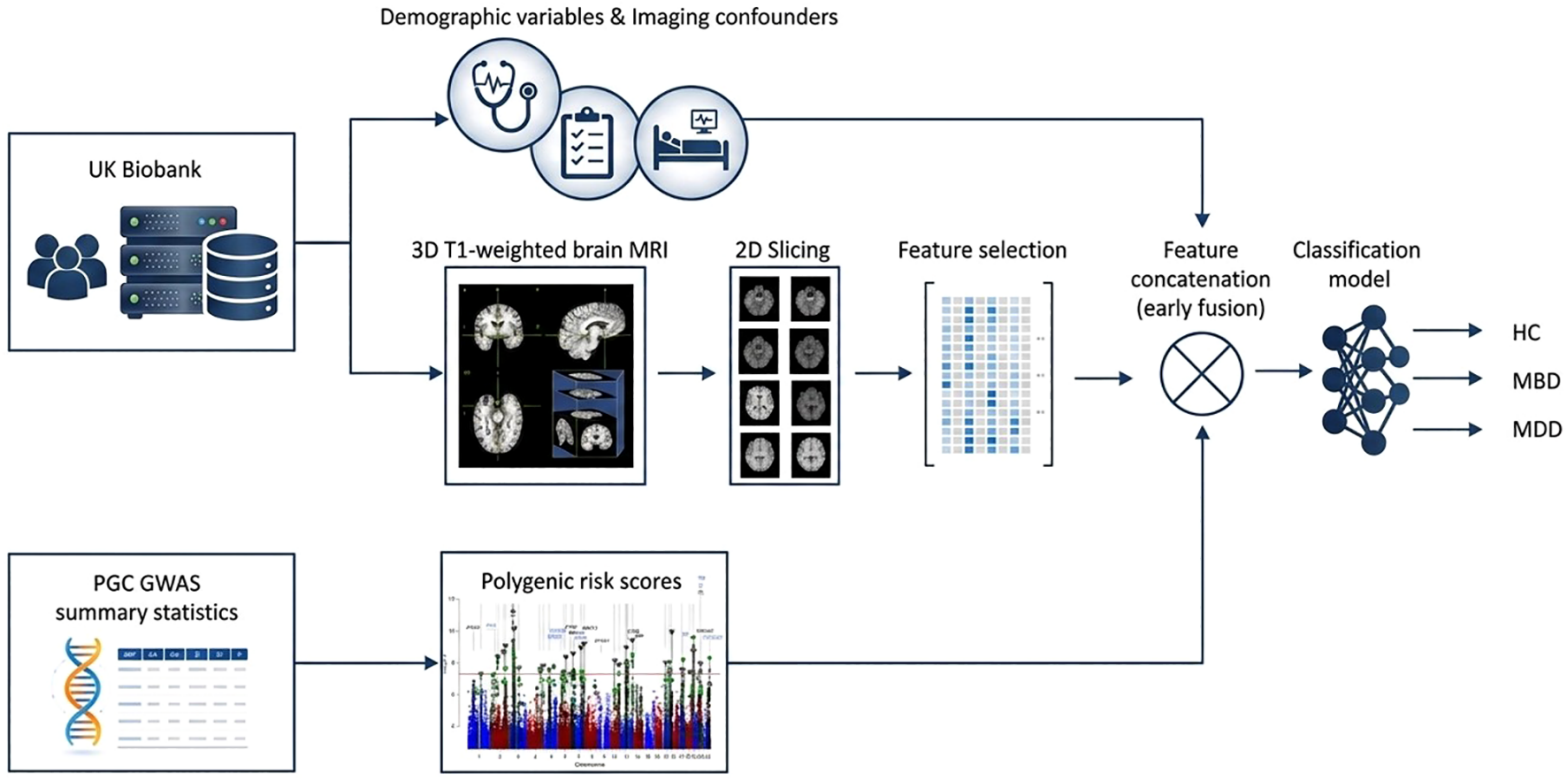
Overall flow of the study.

This study employs a multimodal machine learning framework to classify individuals into three diagnostic categories: healthy controls (HC), mania/bipolar disorder (MBD), and major depressive disorder (MDD), using an early fusion strategy combining structural brain MRI and polygenic risk scores derived from genome-wide association studies. The overall study workflow is illustrated in **Figure 1**. Throughout this manuscript, ‘MBD’ refers specifically to the present study’s UK Biobank-derived diagnostic category, while ‘BD’ and ‘bipolar disorder’ are used when referring to the general clinical condition or to findings from other studies.

### 2.1 Data sources

This study adopts a retrospective, cross-sectional design, utilising previously collected imaging, genetic, and clinical data from the UK Biobank cohort. No new data were collected; all variables were drawn from existing UK Biobank records, with baseline assessments, MRI imaging, and linked health records obtained at different time points for each participant.

UK Biobank is a large-scale general population research cohort comprising more than half a million adults in middle to older age (23). Participants attended baseline assessments between 2006 and 2010, during which blood samples were provided for genetic analyses. Brain imaging was conducted at follow-up visits from 2014 onwards. As of 25 April 2023, a total of 49,335 participants had T1-weighted structural brain images available (HC: 43,220; MBD: 169; MDD: 245; other mood disorders: 5,701). UK Biobank received ethical approval (reference 21/NW/0157) and all participants provided informed consent. The present project was conducted under approved project 71392 (PI Strawbridge).

Variables analysed included 3D T1-weighted MRI images and polygenic risk scores, alongside imaging confounders: age, sex, genetic ethnic grouping, estimated total intracranial volume, head motion during T1 acquisition, and scanner positional coordinates (lateral, transverse, and longitudinal). Imaging processing and genotyping were conducted centrally by UK Biobank and are described elsewhere (24, 25). Categorical confounders (sex, age group, and genetic ethnic grouping) were label-encoded, while continuous imaging confounders (estimated total intracranial volume, head motion, and the three scanner positional coordinates) were Min-Max scaled. All confounders were concatenated directly into the feature matrix alongside the MRI and/or PRS features for each dataset configuration, rather than being statistically regressed out as covariates.

Case selection followed a temporally anchored retrospective approach: individuals with MBD or MDD were included only where the date of first recorded ICD-10 diagnosis (from self-report, primary care, or hospital records; First Occurrences field #1712) preceded the imaging assessment date, ensuring diagnoses were established prior to and independent of the neuroimaging data used as model input. Healthy controls were defined as participants with no mood disorder diagnosis recorded up to the imaging date. This design minimises reverse causation risk, though consistent with retrospective studies generally, it may introduce selection bias toward more clinically established presentations. Stratified sampling by sex, age group, and genetic ethnic grouping was applied to ensure balanced class representation across all three diagnostic categories.

Polygenic risk scores for bipolar disorder (PRS-MBD) and major depressive disorder (PRS-MDD) were derived from GWAS summary statistics obtained from the Psychiatric Genetics Consortium (26, 27), using LDpred with a reference panel of 1,000 European ancestry UK Biobank participants. PRS analyses were restricted to participants of European ancestry to ensure alignment between the derivation GWAS and the target sample. LDpred applies a continuous shrinkage approach across genome-wide variants rather than selecting a fixed subset of variants based on a hard p-value threshold. Therefore, a single fixed variant count is not reported in the same way as threshold-based PRS approaches, as each score reflects the genome-wide set of quality-controlled SNPs available after GWAS summary statistic harmonisation.

### 2.2 Dataset subsets

The dataset comprises three subsets, structured as an ablation study to systematically isolate and evaluate the independent and combined contributions of neuroimaging and genetic data to mood disorder classification as in **Table 1**. Each subset holds equal class sizes across HC, MBD, and MDD, ensuring balanced evaluation conditions throughout.

**Table 1:** Details of the three dataset configurations.

Dataset	Category	Subjects	Gender (M/F)	Age range; Mean ± SD
*D1: MRI + demographics + confounders*	HC	142	69/73	48–80; 64.25 ± 7.74
	MBD	142	58/84	48–80; 62.29 ± 7.10
	MDD	142	54/88	48–79; 62.55 ± 7.41
*D2: PRS + demographics + confounders*	HC	88	45/43	48–77; 64.42 ± 7.29
	MBD	88	36/52	49–78; 62.90 ± 6.42
	MDD	88	35/53	48–79; 62.97 ± 7.18
*D3: MRI + PRS + demographics + confounders*	HC	88	45/43	48–77; 64.42 ± 7.29
	MBD	88	36/52	49–78; 62.90 ± 6.42
	MDD	88	35/53	48–79; 62.97 ± 7.18

D1, Dataset 1; D2, Dataset 2; D3, Dataset 3; MRI, magnetic resonance imaging; PRS, polygenic risk scores; HC, healthy controls; MBD, mania/bipolar disorder; MDD, major depressive disorder; M, male; F, female; SD, standard deviation.

Dataset 1 (D1) comprises 426 participants (142 per class) and includes MRI image features, demographic variables, and imaging confounders. This represents the neuroimaging-only condition, establishing the baseline contribution of structural brain imaging to classification.

Dataset 2 (D2) comprises 264 participants (88 per class) and replaces MRI features with PRS alongside the same demographic and imaging confounders as D1. This represents the genetics-only condition, enabling assessment of whether genetic risk information alone provides meaningful discriminatory signal.

Dataset 3 (D3) comprises 264 participants (88 per class) and combines both MRI features and PRS alongside the same confounders, representing the full multimodal fusion condition. D2 and D3 are drawn from the same pool of 264 participants, making the D2-versus-D3 comparison a controlled, same-sample evaluation of the added value of incorporating MRI into the genetic model. D1 is larger because PRS availability was not required for its construction. Differences in performance between D1 and D2/D3 should therefore be interpreted in the context of both feature composition and sample size, while the D2-versus-D3 comparison provides a sample-size-controlled assessment of MRI feature contribution.

### 2.3 Image preprocessing and slice selection

T1-weighted 3D MRI volumes were preprocessed centrally by UK Biobank (24). MRI slices were initially loaded as 3-channel RGB arrays for compatibility with the image processing pipeline. As T1-weighted MRI encodes a single intensity value per voxel with no colour or spectral information, the three channels contain identical data; slices were therefore converted to single-channel grayscale representations prior to further processing, ensuring the feature space accurately reflects the underlying imaging modality and eliminating redundant duplicate channels.

Shannon entropy was subsequently calculated for each axial slice based on the distribution of grayscale intensity values. For a given axial slice *s* comprising *k* discrete voxel intensity bins, each occurring with probability *p_k_*, the slice-level entropy is defined as:


$$E_{s}=-\sum_{k}p_{k}log_{2}\left(p_{k}\right)$$

where *p_k_* denotes the probability of voxel intensity bin *k* within the slice, and log_2_ is the binary logarithm. Entropy reaches its maximum when all intensity values are distributed uniformly across bins, and approaches zero when the distribution is concentrated at a single value. In the context of structural MRI, slices with high entropy values exhibit heterogeneous voxel intensity distributions, indicative of regions where grey matter, white matter, and cerebrospinal fluid coexist in spatially complex arrangements.

Entropy was computed independently for each axial slice, and slices were subsequently ranked in descending order of their entropy scores. The eight slices with the highest entropy values were retained per participant (19–21). This entropy-guided selection strategy was adopted in preference to fixed anatomical reference positions, which may not consistently capture the most structurally informative regions across individuals owing to inter-subject variability in brain morphology. Each selected slice was resized to 64×64 pixels, yielding a flattened feature vector of 4,096 values per slice. Across eight slices per participant, this produced an initial MRI feature space of 32,768 features prior to feature selection.

### 2.4 Feature selection

Given the high dimensionality of the MRI feature space relative to the available sample size, univariate feature selection was applied using the SelectKBest method from the scikit-learn library, retaining the k features most associated with the target class labels via ANOVA F-test scoring. The value of k was determined empirically by evaluating classification accuracy across a range of k values on the training set; k=150 produced the highest validation accuracy of 66% and was applied consistently across all subsequent models and datasets.

### 2.5 Model development and evaluation

Six machine learning classifiers were trained and evaluated: XGBoost, Light Gradient Boosting Machine (LGBM), Random Forest, Decision Tree, Logistic Regression with elastic net penalty, and Support Vector Machine (SVM). Hyperparameter optimisation was performed for all classifiers using the Optuna framework, with optimised parameters and search ranges summarised in **Table 2**. Optimisation was conducted using stratified 10-fold cross-validation within each Optuna search, with the number of trials scaled to classifier complexity.

**Table 2:** Hyperparameter search ranges for all classifiers optimised using the Optuna framework.

Classifier	Trials	Optimised hyperparameters
LGBM	100–150	lambda_l1 [1e-8, 10.0], lambda_l2 [1e-8, 10.0], num_leaves [2, 256], feature_fraction [0.4, 1.0], bagging_fraction [0.4, 1.0], bagging_freq [1, 7], min_child_samples [5, 100]
XGBoost	60–100	max_depth [1, 9], learning_rate [0.01, 1.0], n_estimators [50, 500], min_child_weight [1, 10], gamma [1e-8, 1.0], subsample [0.01, 1.0], colsample_bytree [0.01, 1.0], reg_alpha [1e-8, 1.0], reg_lambda [1e-8, 1.0]
Random Forest	50	n_estimators [100, 500], max_depth [10, 110], min_samples_split [2, 10], min_samples_leaf [1, 4]
Logistic Regression	10–50	l1_ratio [0, 1], C [1e-10, 1000]; penalty = elasticnet, solver = saga, max_iter = 5,000
SVM	100	C [1e-2, 1e2], kernel [linear, poly, rbf]; gamma = 0.01 (fixed)
Decision Tree	100	max_depth [2, 64]

Data were partitioned into training and test sets using a stratified 80:20 split prior to any model fitting or hyperparameter search. Preprocessing steps requiring fitted parameters, including feature selection and variable scaling, were fitted on the training data and then applied to the held-out test data. The same stratified 10-fold StratifiedKFold configuration with a fixed random state was applied consistently across all classifiers to ensure reproducibility and fair comparison (28). Final models were trained using the best identified hyperparameters on the full training set and evaluated on the held-out test set. All classifiers were trained and evaluated independently on each of the three dataset configurations.

Model performance was evaluated using macro-averaged AUC as the primary performance metric, given the three-class task and balanced class distribution (29, 30). Per-class AUC values are additionally reported to characterise classification performance at the individual diagnostic category level.

## 3 Results

### 3.1 Entropy ranking and slice selection

**Figure 2:**
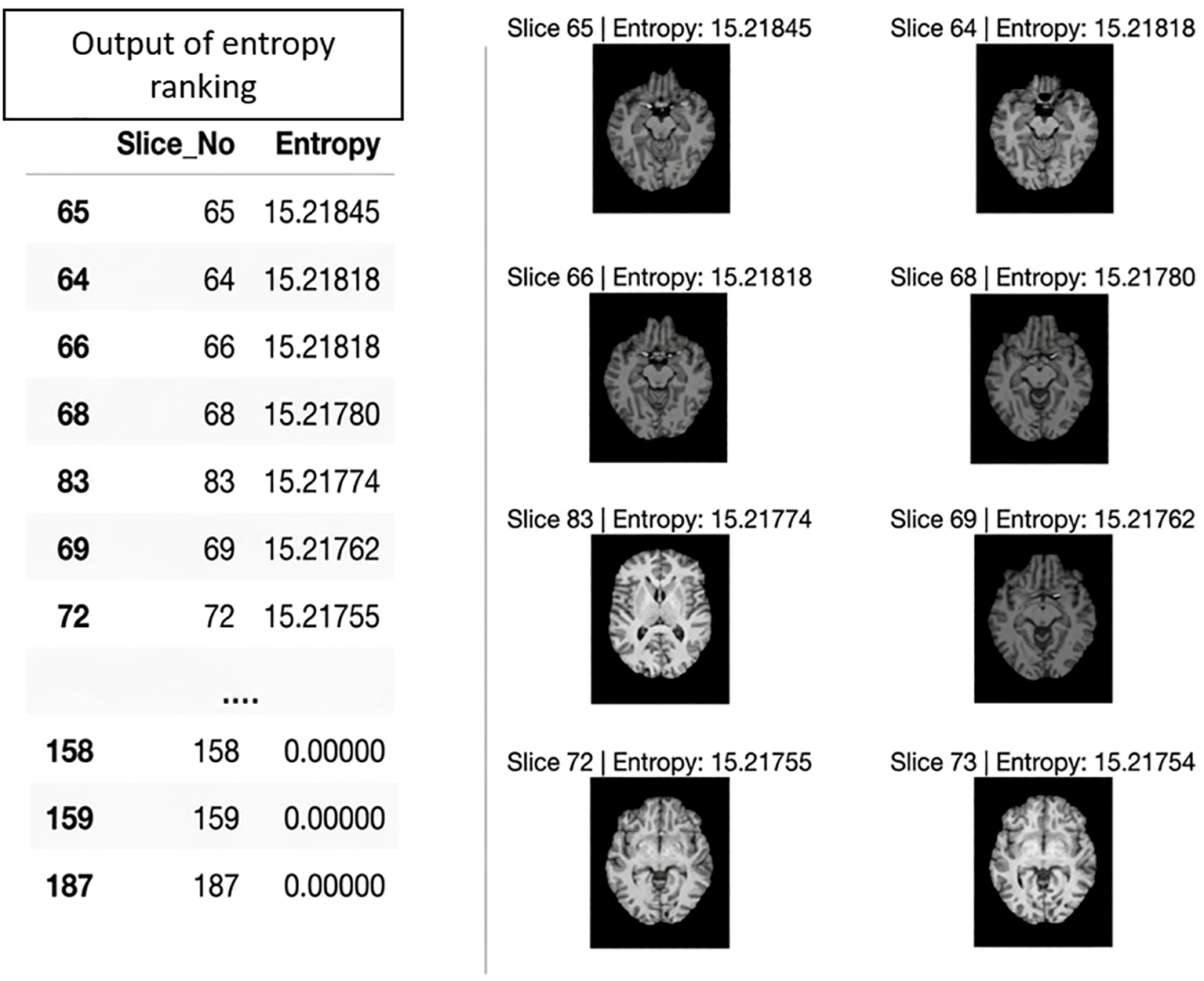
Example of output from entropy ranking and eight axial 2D slices extracted. Reproduced from UK Biobank with permission.

The entropy ranking procedure was applied independently to each axial slice across all participants, producing a ranked ordering of slices from highest to lowest information content based on the distribution of grayscale intensity values. Slices with the highest entropy scores correspond to brain regions exhibiting the greatest voxel intensity diversity, reflecting the spatial coexistence of grey matter, white matter, and cerebrospinal fluid in structurally complex arrangements. An example of the entropy-ranked output and the eight axial slices retained per participant is illustrated in **Figure 2**. The selected slices consistently captured cortical and subcortical regions rather than peripheral or largely homogeneous areas such as skull boundary slices, demonstrating that the entropy-guided approach successfully oriented slice selection toward the most structurally informative portions of the MRI volume.

### 3.2 Feature selection of images

**Figure 3:**
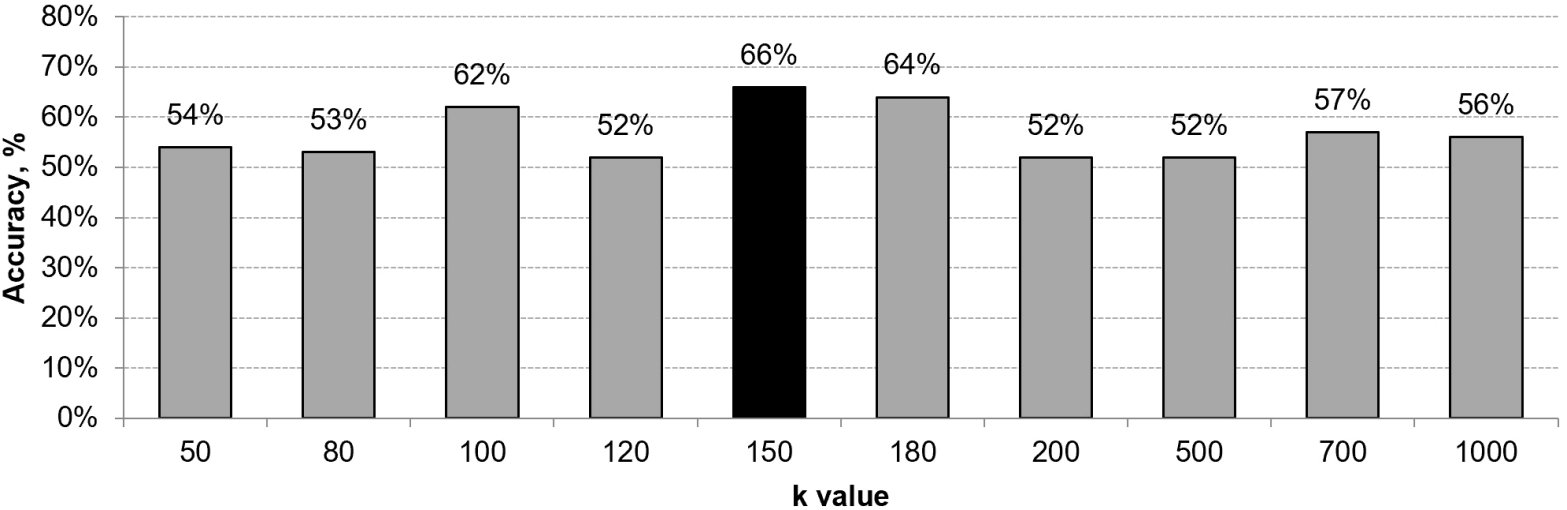
The results of testing the SelectKBest method using different k values.

Feature selection was applied to the MRI feature space using SelectKBest with ANOVA F-test scoring. The effect of varying k on validation accuracy is illustrated in **Figure 3**. Classification accuracy increased with k up to a point before stabilising or declining at higher values, indicating that beyond a threshold, additional features introduced noise that reduced generalisation performance. k=150 yielded the highest validation accuracy of 66% and was adopted for all subsequent models and datasets.

### 3.3 Performance of classification models

The performance of all classifiers across the three datasets is summarised in **Table 3**. AUC is treated as the primary evaluation metric given the three-class nature of the task and the real-world clinical dataset.

**Table 3:** ROC-AUC values for all classifiers across datasets and diagnostic categories.

	Dataset 1 (D1)				Dataset 2 (D2)				Dataset 3 (D3)			
Model	HC	MBD	MDD	Macro	HC	MBD	MDD	Macro	HC	MBD	MDD	Macro
LGBM	0.7753	0.7295	0.7729	0.7592	0.7099	0.7323	0.6335	0.6919	0.6790	0.7855	0.6528	0.7059
XGBoost	0.7527	0.7241	0.8175*	0.7647*	0.6744	0.7160	0.5895	0.6600	0.6867	0.7840	0.6651	0.7121
Random Forest	0.7491	0.7461	0.7527	0.7493	0.6559	0.6759	0.5802	0.6374	0.5880	0.6836	0.5772	0.6163
Decision Tree	0.5603	0.6385	0.6626	0.6205	0.7361	0.5579	0.6674	0.6538	0.6277	0.5802	0.4552	0.5561
Logistic Regression	0.6284	0.7378	0.7039	0.6900	0.6049	0.5741	0.5880	0.5892	0.6188	0.6034	0.6991	0.6405
SVM	0.6908	0.7586	0.7408	0.7301	0.6003	0.5586	0.6019	0.5870	0.7068	0.7531	0.6620	0.7075

*Highest AUC observed across all models and datasets (XGBoost, D1, MDD-specific AUC = 0.8175; macro-average AUC = 0.7647). AUC, area under the receiver operating characteristic curve; HC, healthy controls; MBD, mania/bipolar disorder; MDD, major depressive disorder; LGBM, light gradient boosting machine; SVM, support vector machine.

The bold/asterisked values indicate the highest AUC values observed across all models and datasets: MDD-specific AUC = 0.8175 and macro-average AUC = 0.7647, both achieved by XGBoost on Dataset 1.

XGBoost demonstrated the strongest overall performance across all datasets and metrics. On D1, XGBoost achieved an AUC of 0.8175 for MDD classification and a macro-average AUC of 0.7647, the peak performance observed across all conditions in this study. LGBM followed closely with a macro-average AUC of 0.7592 on D1, with per-class AUC values of 0.7753 for HC, 0.7295 for MBD, and 0.7729 for MDD. Random Forest achieved a macro-average AUC of 0.7493 on D1, with closely balanced per-class performance across HC (0.7491), MBD (0.7461), and MDD (0.7527). SVM achieved a macro-average AUC of 0.7301 on D1, with its strongest per-class performance observed for MBD (0.7586). Logistic Regression and Decision Tree returned lower macro-average AUC values of 0.6900 and 0.6205 respectively on D1, with Decision Tree recording the lowest HC AUC of 0.5603 across all classifiers and datasets.

Across all classifiers on D1, MBD consistently returned lower AUC values relative to HC and MDD, with per-class MBD AUC ranging from 0.6385 for Decision Tree to 0.7586 for SVM. MDD classification on D1 showed the greatest variability across classifiers, with XGBoost achieving the highest MDD-specific AUC of 0.8175 while Decision Tree returned 0.6626.

Performance declined across most classifiers when moving from D1 to D2. XGBoost macro-average AUC dropped from 0.7647 on D1 to 0.6600 on D2, while LGBM dropped from 0.7592 to 0.6919 and Random Forest from 0.7493 to 0.6374. MDD classification was particularly affected on D2, with XGBoost recording an MDD-specific AUC of 0.5895 and Random Forest recording 0.5802. An exception to the general decline was observed for Decision Tree, which achieved its highest macro-average AUC of 0.6538 on D2, driven by an HC AUC of 0.7361, its strongest per-class result across all configurations. SVM also showed marked deterioration on D2, with macro-average AUC falling to 0.5870 from 0.7301 on D1, and MBD AUC declining to 0.5586.

On D3, performance recovered relative to D2 across most classifiers. XGBoost achieved a macro-average AUC of 0.7121 on D3, an improvement of 0.052 over D2, with MBD AUC increasing from 0.7160 to 0.7840. LGBM recorded a macro-average AUC of 0.7059 on D3, with its highest MBD AUC of 0.7855 observed across all three configurations. SVM recovered to a macro-average AUC of 0.7075 on D3, with MBD AUC returning to 0.7531. Logistic Regression showed modest improvement on D3 relative to D2, with macro-average AUC increasing from 0.5892 to 0.6405 and MDD AUC improving from 0.5880 to 0.6991. Decision Tree continued to decline on D3, recording its lowest macro-average AUC of 0.5561 and an MDD-specific AUC of 0.4552, the lowest value observed across all classifiers and configurations in this study. Random Forest also declined further on D3 relative to D2, with macro-average AUC falling to 0.6163.

### 3.4 Contribution of individual modalities and fusion

A central aim of this study was to evaluate whether combining neuroimaging and genetic data improves mood disorder classification beyond what either modality achieves independently. The three dataset configurations function as a structured ablation, and **Table 3** summarises XGBoost AUC performance across them.

Across all six classifiers, D1 consistently achieved the highest classification performance, confirming that structural MRI carries the strongest discriminatory signal among the modalities evaluated. XGBoost on D1 attained a macro-average AUC of 0.7647 with MDD-specific AUC of 0.8175, suggesting that morphological brain features captured by T1-weighted MRI provide meaningful and replicable signal for distinguishing mood disorder categories in a population cohort.

D2, the genetics-only configuration, produced substantially lower AUC values across all classifiers. XGBoost on D2 achieved a macro-average AUC of 0.6600, indicating that PRS as currently derived do not provide sufficient standalone discriminatory power for individual-level mood disorder classification. This is consistent with the known modest effect sizes of common genetic variants for complex psychiatric traits at the individual level.

The most methodologically controlled comparison in this study is between D2 and D3, which share an identical sample of 264 participants and differ only in the inclusion of MRI features. XGBoost on D3 achieved a macro-average AUC of 0.7121 against 0.6600 on D2, an improvement of 0.052, with the most pronounced gain in MBD classification (AUC 0.7840 vs 0.7160). This same-sample comparison provides direct evidence that MRI features contribute meaningfully to classification performance beyond what PRS alone achieves.

D3 also attained competitive performance given its smaller sample size (264 vs 426), suggesting complementary information from PRS, but direct comparison with D1 is limited by sample composition. The true benefit of multimodal fusion may be underestimated here given the constrained number of participants with both MRI and PRS data available.

## 4 Discussion

This study evaluated a fusion-based machine learning framework for the simultaneous three-class classification of healthy controls, mania/bipolar disorder, and major depressive disorder using structural brain MRI, polygenic risk scores, and demographic data from the UK Biobank. The three dataset configurations, structured as a controlled ablation study, provide a layered picture of how neuroimaging and genetic data each contribute to classification performance and what their combination achieves relative to either modality alone.

Structural brain MRI emerged as the dominant contributor to classification performance across all conditions evaluated. XGBoost trained on neuroimaging features achieved a macro-average AUC of 0.7647 and an MDD-specific AUC of 0.8175, the highest values observed across all models and dataset configurations in this study. Ensemble gradient boosting methods, specifically XGBoost and LGBM, consistently outperformed simpler classifiers across all three datasets, which is consistent with their known capacity to model complex, non-linear interactions in high-dimensional tabular feature spaces. Random Forest also performed reasonably on D1 with a macro-average AUC of 0.7493, while Decision Tree, Logistic Regression, and SVM showed comparatively weaker and less consistent discriminatory performance. Decision Tree in particular exhibited erratic behaviour across configurations, attaining a macro-average AUC of 0.6538 on D2 but collapsing to 0.5561 on D3 despite the latter containing additional MRI features, suggesting instability in the presence of high-dimensional heterogeneous input. SVM demonstrated a distinct pattern, performing comparably to ensemble methods on D1 and D3 but substantially weakening on D2, indicating differential sensitivity to feature type that warrants further investigation. This instability reinforces the preference for ensemble methods, such as XGBoost and LGBM, over single-tree models when handling high-dimensional, heterogeneous multimodal data.

The aggressive dimensionality reduction from 32,768 to 150 features, representing a 99.5% reduction via ANOVA F-test univariate selection, merits consideration. While k=150 was determined empirically based on validation accuracy, such extreme compression necessarily discards a large proportion of the pixel-level feature space. This reflects a fundamental limitation of raw pixel-based MRI representations relative to derived quantitative phenotypes, where features such as cortical thickness or voxel-based morphometry measures encode anatomically meaningful variation more efficiently. The competitive performance achieved despite this compression suggests that entropy-guided slice selection successfully concentrates diagnostically relevant signal into a manageable subset of voxels, though the ceiling imposed by raw pixel features likely constrains overall classification performance relative to what morphometric approaches might achieve in the same framework. The selection of eight slices was guided by prior entropy-based MRI studies, but future work should examine whether alternative slice numbers affect classification performance. It should also be noted that ANOVA F-test univariate feature selection assumes approximately normally distributed features within each class and homogeneity of variance across classes, assumptions that may not be fully met by raw pixel intensities. Additionally, as a univariate method, it evaluates each feature independently of others, which does not account for the spatial correlation among neighbouring pixels within MRI slices and may affect the optimality of the selected feature subset. This highlights the need for future work to explore feature selection methods that explicitly account for spatial structure, such as region-based or multivariate approaches.

The macro-average AUC of 0.7647 achieved by XGBoost on D1 compares favourably with prior population-level mood disorder classification studies. While studies such as Li et al. (2020) reported 87.5% accuracy for bipolar disorder classification using voxel-based morphometry and regional homogeneity features with SVM (11), and He et al. (2025) achieved 91.2% accuracy distinguishing BD from MDD using structural MRI morphometric features (13), these comparisons are not directly equivalent. Binary case-control discrimination in curated clinical samples is a substantially easier problem than simultaneous three-class classification in a general population cohort where diagnostic effect sizes are smaller and demographic heterogeneity is greater. The MDD-specific AUC of 0.8175 on D1 compares more favourably with prior MDD-focused studies; Winter et al. (2024) reported a maximum balanced accuracy of 62.0% for MDD classification from derived MRI and DTI features across multiple modalities (31), and Poirot et al. (2026) achieved an AUC of 0.57 from grey matter alone in nearly 5,000 UK Biobank participants (18), both of which contextualise the present findings as competitive for population-level mood disorder classification using neuroimaging.

MBD consistently represented the most difficult diagnostic category to classify across all datasets and classifiers, a pattern that reflects the known clinical and neurobiological overlap between bipolar disorder and major depressive disorder, particularly during depressive episodes. The original UK Biobank cohort contained only 169 MBD cases and 245 MDD cases against 43,220 healthy controls, underscoring the extreme natural class imbalance in population-based psychiatric research. While stratified sampling ensured balanced class representation across all three diagnostic categories in the experimental datasets, the small absolute numbers of mood disorder cases, particularly MBD, constrain the diversity of presentations available for model training and likely contribute to the difficulty in achieving reliable MBD discrimination. This is a structural challenge inherent to population cohort studies of psychiatric conditions rather than a limitation specific to the modelling approach adopted here.

PRS alone provided limited individual-level discriminatory signal for mood disorder classification. Models trained on the genetics-only configuration produced consistently lower AUC values across all classifiers and diagnostic categories, with XGBoost achieving a macro-average AUC of 0.6600, reflecting the modest effect sizes of individual polygenic variants for complex psychiatric conditions. It should be noted that D1 and D2 differ in both feature composition and sample size, which limits their direct comparison. However, the direction of the performance difference is unlikely to be attributable to sample size alone, given that D3 at the same 264-participant size achieves considerably stronger performance through the addition of MRI features.

From a translational standpoint, the modest AUC improvement of 0.052 observed when adding PRS to the multimodal model (D2 to D3) raises a legitimate question as to whether this incremental gain justifies the cost and logistical burden of systematic genotyping in routine clinical practice. Genome-wide genotyping remains costly and is not yet standard in psychiatric diagnostic workflows, and the marginal classification benefit demonstrated here would need to be weighed against these practical constraints. However, several factors suggest this calculus may shift favourably over time: genotyping costs continue to decline, many patients already undergo genotyping for other clinical or research purposes, allowing PRS to be calculated at negligible marginal cost, and the value of PRS may be greater in settings where neuroimaging is unavailable or impractical, such as primary care. At present, PRS may be more readily justified as a research tool for understanding disease mechanisms and stratifying study populations than as a standalone driver of clinical genotyping decisions, with its clinical utility likely to be realised primarily in combination with other low-cost, scalable data sources rather than in isolation.

The most methodologically controlled and informative comparison in this study is between D2 and D3, which share an identical sample of 264 participants and differ only in the inclusion of neuroimaging features. The improvement in macro-average AUC from 0.6600 to 0.7121 for XGBoost, with the most pronounced gain observed in MBD classification (AUC 0.7160 to 0.7840), provides direct evidence that MRI features contribute discriminative value beyond what PRS alone achieves. A broadly similar pattern was reported by Lee et al. (2024), where MRI-genetic fusion substantially outperformed genetics-only models for three-class mood disorder classification with an AUC improvement exceeding 0.21 (16), although their use of whole-exome sequencing, deep learning architectures, and a clinical rather than population-based sample makes direct performance comparison difficult. This should therefore be interpreted as supportive, rather than definitive, evidence that PRS may provide complementary information to structural MRI in a smaller matched sample. The true benefit of multimodal fusion may be further underestimated here given the constrained number of participants with both MRI and PRS data available, and larger matched samples would be expected to yield more pronounced fusion gains.

Taken together, these findings indicate that structural MRI and PRS provide complementary rather than redundant information for mood disorder classification. Neither modality alone achieves the discriminatory performance of their combination in a same-sample controlled setting, and the practical implication for future multimodal psychiatric classification studies is that deliberate integration of imaging and genetic modalities may yield more generalisable models than either source independently, particularly as larger datasets with matched imaging and genomic data become available.

Several limitations of this study warrant consideration. The restricted availability of MBD and MDD cases within UK Biobank resulted in relatively small sample sizes, particularly for D2 and D3, constraining generalisability and limiting statistical power for detecting subtle classification differences. Now that UK Biobank have reached their target of completing MRI scanning of 100,000 participants, revisiting this analysis with larger samples would be a valuable next step. PRS analyses were restricted to participants of European ancestry due to the requirement for ancestry matching between the derivation GWAS and the target sample; the absence of comparably powered GWAS studies for BD and MDD in non-European ancestries currently precludes extending this approach more broadly, though this represents an important direction as diverse genetic resources continue to expand. External validation on an independent cohort was not possible within the scope of this study, and confirmation of the generalisability of the learned classification patterns on a separate dataset remains necessary. This study also used raw pixel-level MRI features rather than derived quantitative phenotypes such as cortical thickness or voxel-based morphometry measures, and whether such derived features would yield superior classification performance within this fusion framework is an open question that merits investigation.

AUC was used throughout as a descriptive metric for comparing and selecting among the six classifiers evaluated, rather than as part of a confirmatory hypothesis-testing framework. No formal statistical correction for multiple comparisons was applied across these comparisons, and this is acknowledged for transparency. Future replication studies should incorporate corrected pairwise statistical testing where formal inferential claims regarding classifier superiority are intended. Additionally, the initial 80:20 train/test partition was not fixed by a random seed, meaning a different partition would be obtained on repeated runs; while the subsequent 10-fold cross-validation used a fixed random state for comparability across classifiers, repeated train/test splits would provide a more robust estimate of model stability.

## 5 Conclusion

In summary, this study proposed and evaluated a fusion-based machine learning framework for three-class mood disorder classification using multimodal data from the UK Biobank, yielding three principal conclusions. Structural brain MRI represents the strongest single contributor to classification performance, with XGBoost achieving an AUC of 0.8175 for MDD classification and a macro-average AUC of 0.7647, comparing favourably with prior MDD classification studies using derived MRI phenotypes. PRS alone provide limited individual-level discriminatory signal, with models trained without neuroimaging data showing consistently lower AUC across all classifiers and diagnostic categories, reflecting the modest effect sizes of common genetic variants for complex psychiatric conditions. Multimodal fusion demonstrates clear added value in a controlled same-sample comparison, improving macro-average AUC from 0.6600 to 0.7121 for XGBoost relative to the PRS-only configuration, with the most pronounced gain in MBD classification and competitive performance despite the smaller sample size. Future work should prioritise external validation on an independent cohort, expansion of PRS to additional psychiatric phenotypes, and investigation of whether derived quantitative MRI features outperform the raw pixel-based approach adopted here. More broadly, these findings support the continued development of computational psychiatry approaches for improving data-driven differential diagnosis between MDD and BD. As UK Biobank participants return for repeat imaging visits, longitudinal modelling of mood disorder onset among currently healthy participants represents a particularly promising direction for future research.

## Data Availability

The original contributions presented in the study are included in the article/supplementary material. Further inquiries can be directed to the corresponding author.
